# Chaperone-based procedure to increase yields of soluble recombinant proteins produced in *E. coli*

**DOI:** 10.1186/1472-6750-7-32

**Published:** 2007-06-12

**Authors:** Ario de Marco, Elke Deuerling, Axel Mogk, Toshifumi Tomoyasu, Bernd Bukau

**Affiliations:** 1 EMBL Heidelberg, Meyerhofstrasse 1, D-69117 Heidelberg, Germany; 2 IFOM-IEO Campus for Oncogenomics, via Adamello 16, I-20139, Milano, Italy; 3 ZMBH, Universität Heidelberg, Im Neuenheimer Feld 282, D-69120 Heidelberg, Germany; 4 Department of Microbiology and Molecular Genetics, Graduate School of Pharmaceutical Sciences, Chiba University, 1-33 Yayoicho, Inageku, Chiba 263-8522, Japan

## Abstract

**Background:**

The overproduction of recombinant proteins in host cells often leads to their misfolding and aggregation. Previous attempts to increase the solubility of recombinant proteins by co-overproduction of individual chaperones were only partially successful. We now assessed the effects of combined overproduction of the functionally cooperating chaperone network of the *E. coli *cytosol on the solubility of recombinant proteins.

**Results:**

A two-step procedure was found to show the strongest enhancement of solubility. In a first step, the four chaperone systems GroEL/GroES, DnaK/DnaJ/GrpE, ClpB and the small HSPs IbpA/IbpB, were coordinately co-overproduced with recombinant proteins to optimize *de novo *folding. In a second step, protein biosynthesis was inhibited to permit chaperone mediated refolding of misfolded and aggregated proteins *in vivo*. This novel strategy increased the solubility of 70% of 64 different heterologous proteins tested up to 42-fold.

**Conclusion:**

The engineered *E. coli *strains and the two-step procedure presented here led to a remarkable increase in the solubility of a various recombinant proteins and should be applicable to a wide range of target proteins produced in biotechnology.

## Background

Chaperones assist the folding of newly synthesized proteins to the native state and provide a quality control system that refolds misfolded and aggregated proteins. In the *E. coli *cytosol, the folding of newly synthesized proteins is assisted by the ribosome-associated Trigger Factor, the DnaK system (DnaK with its DnaJ and GrpE cochaperones; KJE), and the GroEL system (GroEL with its GroES cochaperone; ELS) [1-4]. KJE and ELS also prevent aggregation and promote refolding of preexisting proteins which lost their native conformation e.g. due to thermal denaturation or intrinsic instability [5,6]. KJE furthermore cooperates with ClpB to solubilize aggregated proteins and refold them to the native state in a concerted action with ELS [7-9]. Moreover, the small heat shock proteins (sHSP) of *E. coli*, IbpA and IbpB (IbpAB), intercalate into protein aggregates and thereby facilitate the KJE/ClpB dependent disaggregation and refolding [10-12].

The overproduction of recombinant proteins in host cells often leads to their misfolding and aggregation [13-17]. Folding problems of overproduced client proteins can be caused by limitations in the chaperone capacity of the host cells. Several attempts were made to increase the yields of correctly folded, and hence soluble, recombinant proteins by the co-overproduction of individual chaperones in producing cells, however only with a limited success. For example, the co-overproduction of ELS increases the solubility of human ORP150, human lysozyme, p50^csk ^protein tyrosine kinase, phosphomannose isomerase and fusion protein PreS2-S'-β-galactosidase and maize protoporphyrinogen IX oxidase [18-23]. The co-overproduction of KJE increases the solubility of endostatin, human ORP150, transglutaminase and PreS2-S'-β-galactosidase [21,24,25].

To assess the full potential of the cellular network of molecular chaperones for the production of soluble recombinant proteins we performed a systematic analysis of the combined power of the major cytosolic chaperone systems of *E. coli*, KJE, ELS, ClpB and IbpAB. We did not include Trigger Factor in our analyses since it acts through a 1:1 association with ribosomes and is already present in three-fold molar excess over ribosomes in wild type cells [2].

## Results and Discussion

**Figure 1:**
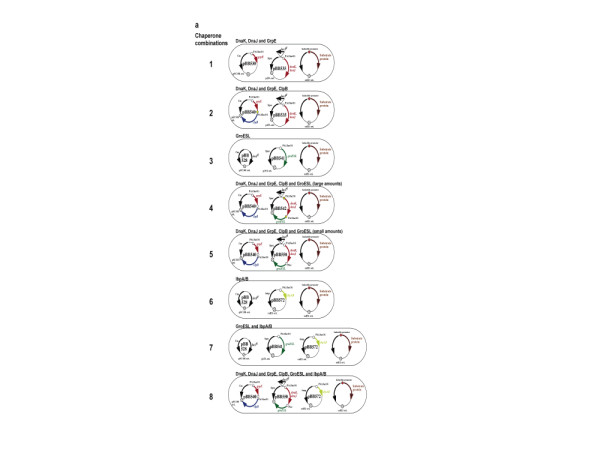
**Design of *E. coli *host cells for the simultaneous overproduction of recombinant target proteins and major chaperone systems**. Host cells for recombinant protein expression containing combinations (1 to 8) of two or three chaperone expression plasmids. Each combination of plasmids includes one plasmid carrying the *lacI*^*Q *^gene to allow for IPTG controlled chaperone expression.

We first investigated a combination of the two major chaperone systems with folding activity, KJE and ELS, and the co-operating chaperone with disaggregating activity, ClpB. To allow for regulated overproduction of chaperones we generated a set of compatible plasmids, which (i) differ in copy number (pSC101, 3–4 copies/cell; p15A, 20–30 copies/cell), (ii) express chaperone genes under control of a strong (PA1) or weaker (Plac) promoter combined with the lacO1 operator (PA1/lacO1) [26], and (iii) encode the *lacI*^*q *^repressor gene. This set of plasmids was designed to allow for IPTG-inducible expression of chaperone genes in different combinations (Fig. 1, combinations 1 to 5), and to produce chaperones at the stoichiometries, which are optimal for their folding activity [27,28].

**Figure 2:**
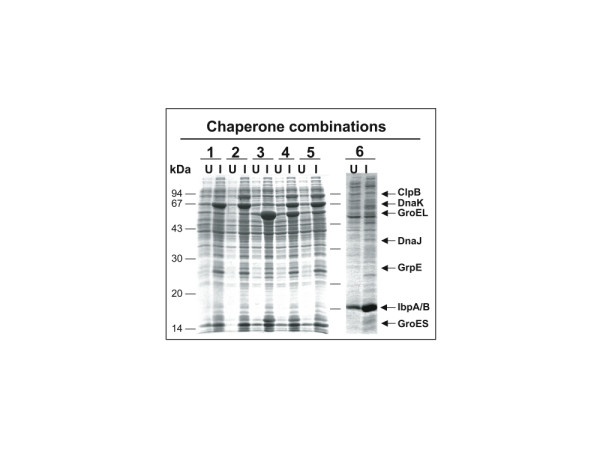
**Chaperone overproduction upon IPTG induction in *E. coli *cells carrying plasmid combinations 1 to 6**. Lysates of non-induced cells (U) and IPTG-induced cells (I) grown overnight at 20°C were separated by SDS-PAGE and Coomassie-stained. The various plasmid combinations present in the tested cells are indicated.

Host cell were transformed with plasmids in 5 combinations. Combination 1 (pBB530 and pBB535) for overproduction of KJE; combination 2 (pBB540 and pBB535) for overproduction of KJE and ClpB; combination 3 (pBB528 and pBB541) for overproduction of ELS; combination 4 (pBB540 and pBB542) for overproduction of KJE, ClpB and high amounts of ELS; combination 5 (pBB540 and 550) for overproduction of KJE with ClpB and lower amounts of ELS (Fig. 1). Continuous growth of these cells in medium containing 100 μM IPTG resulted in an increase of DnaK (18–22 fold), ClpB (15–18 fold), GroEL (30 fold, combination 4; 5 fold, combination 5) over wild type levels (Fig. 2), without causing apparent growth defects (data not shown). Host cells containing different combinations of these plasmids were subsequently transformed with plasmids expressing 50 different recombinant genes of prokaryotic and eukaryotic origin (Table 1) from IPTG-controlled promoters. The encoded proteins include monomers and oligomers, cytosolic, membrane bound and secreted proteins, full-length, fragmented and fused proteins (fusion to GST, Trx, DsbA or NusA) [see Additional file 1], with molecular weights ranging between 7.5 and 118 kDa. Proteins were hexa-histidine tagged to allow affinity purification of the soluble fractions.

**Figure 3:**
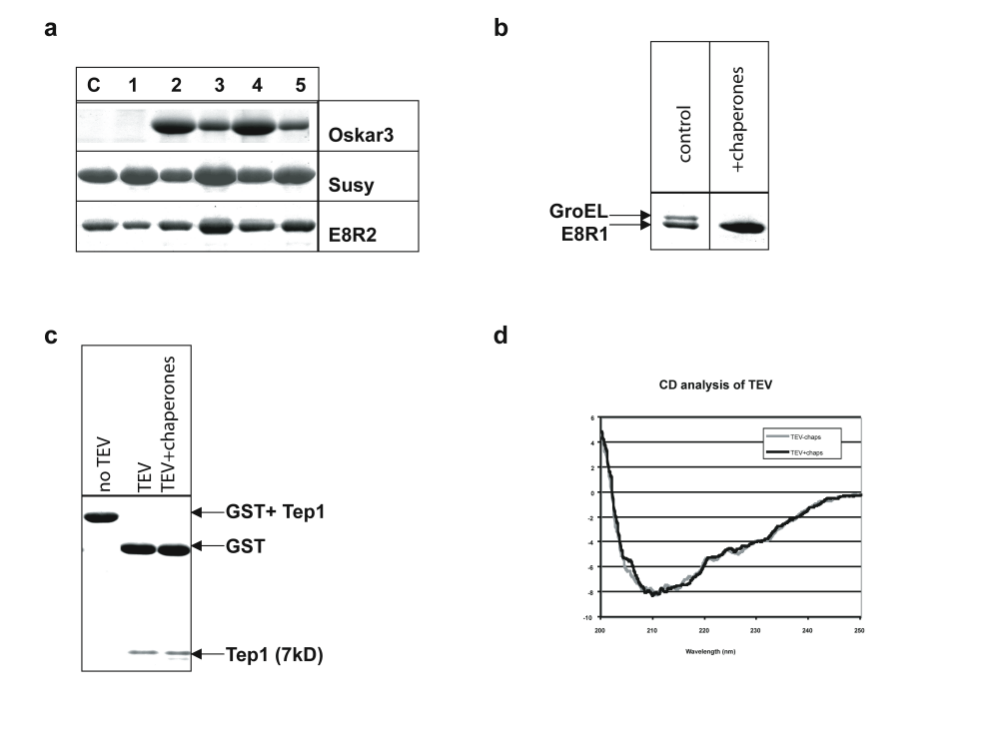
**Effects of the chaperone co-expression on the yields of soluble target proteins**. (a) Coomassie-stained SDS-PAGE showing target proteins purified by metal affinity chromatography either from control cells (C; without chaperone overexpression) or from different host cells overexpressing chaperone combinations 1 to 5 (indicated by the numbers above the lanes). The proteins Oskar3, Susy, and E8R2 were purified from cells grown with the one-step protocol. (b) Chaperone over-expression (combination 5) in host cells prevents the co-elution of the target protein E8R1 together with GroEL. (c) Comparison of the TEV activity using TEV protease purified from wild type cells and chaperone over-expressing cells (combination 3). (d) CD analysis of the TEV protein purified from wild type cells without (gray line) and with chaperone overexpression (combination 3, black line).

26 of the 50 target proteins tested showed an increase in the final yield of purified, soluble protein upon co-overproduction of chaperones (Table 1; see Fig. 3a for examples). Of these 26 proteins, 21 showed a 2.5- to 5.5-fold increase in solubility, and 5 proteins (e.g. Lzip and Oskar3) became soluble only upon chaperone co-overproduction, allowing its purification under native conditions. For 16 of the 26 target proteins the highest increase in solubility was achieved by co-overproduction of all three KJE, ELS and ClpB chaperone systems in combination 4 or 5, with a higher success rate (11 of 26) for combination 4, which produces higher levels of ELS. For 8 of the 26 proteins the highest increase in solubility was obtained with combination 3, which overproduces ELS alone, and in only one case each the combination 1 (KJE alone) or 2 (KJE and ClpB) yielded the highest degree of solubility of the substrate protein. Taken together, these results show that chaperone co-overproduction is successful in about 50% of the proteins tested, with KJE, ClpB and ELS being the most successful combination. We did not observe any influence of the protein fusion tags, which have been fused to some of the recombinant proteins, on the efficiency of chaperone mediated solubility increase [see Additional file 1]. Furthermore, identical beneficial effects of chaperone co-production were observed when selected recombinant proteins (Btke, 2Tep4, Susy, Oskar1, TEV, Xklp3A/B) were produced in larger culture volumes (1–10 L) with final yields in the range of 2–20 mg/L [29].

We tested for several recombinant proteins whether their solubility reflects the folding to the native state. TEV protease purified from the soluble fractions of cells with and without co-overproduction of ELS was active in cleaving a GST-Tep1 domain fusion protein (Fig. 3c) and showed highly similar circular dichroism spectra (Fig. 3d). For the target protein E8R1 the soluble material purified from cells without chaperone co-overproduction showed co-purification of significant amounts of EL, in contrast to the E8R1 protein purified from ELS/KJE/ClpB overproducing cells which appears EL-free (Fig. 3b), indicative of correct folding. These results suggest that the overproduction of chaperones not only results in improved solubility of the recombinant proteins tested but also enhances the acquisition of the native state.

**Table 1:** List of proteins co-expressed without and with chaperone combinations 1 to 5 and effects of chaperones on yields of purified, soluble target proteins, applying either the one-step or two-step procedure.

Protein	MW (kDa)	Increased solubility by			
		
		one step procedure		two step procedure	
		
		Yield increase	Best chaperone combination	Yield increase	Best chaperone combination
1Fringe	43	∞	4	3	4
2Ap	54	∞	4	3	3
22f21	52	∞	3	3	4
LZip	41	∞	1	n.d.	n.d.
Oskar3	118	∞	2/4	2.5	2
2c18	50	3	4	8	4
2Tep4	68	3.5	4	13	4
BtKe	55	7	3	42	4
PhK	29	3	4	7	5
Tep1	8	3	5	6	1
Susy	86	2.5	3	5	5
Xklp3A+Xklp3B	20+ 16	2.5	4	3.5	4
B1R	59	3.5	3	3.5	3
E8R2	85	5.5	3	5.5	3
GTR1	35	3	4	3	4
HbpH	9	3.5	4	3.5	4
Rolled	43	4.5	3	4.5	3
Xklp3A4	44	2.5	5	2.5	5
Xklp3B3	70	2.5	4	2.5	4
Mash+ Susy	90+ 86	3	4	3	4
1Tep3	47	4	3	n.d.	n.d.
1Tep4	45	3.5	5	n.d.	n.d.
BtKc	64	3	5	n.d.	n.d.
E8R1	58	5	5	n.d.	n.d.
MaxF	7.5	3	4	n.d.	n.d.
Tev prot.	30	3.5	3	n.d.	n.d.
Xklp3A1	62	1*	-	∞	2
Xklp3B1	40	1*	-	∞	5
2Fringe	55	1	-	2	4
Xklp3A5	35	1	-	19	4
2Tep3	70	1	-	11	2
BtKp	55	1	-	28	4
Mash	91	1	-	3	2
PPAT	22	1	-	3	4
Eg5	95	1	-	1	-
F10L	72	1	-	1	-
Tep2	11	1	-	1	-
1AP	52	1	-	n.d.	n.d.
Chip	64	1	-	n.d.	n.d.
dLMO	37	1	-	n.d.	n.d.
Pex5P	38	1	-	n.d.	n.d.
Endostatin	42	1*	-	1*	-
Kringle	30	1*	-	1*	-
Lzip2	37	1*	-	1*	-
UCP1	33	1*	-	1*	-
Tlc4	57	1*	-	n.d.	n.d.
Xklp3A2	38	1*	-	n.d.	n.d.
Xklp3A3	72	1*	-	n.d.	n.d.
Xklp3B1	35	1*	-	n.d.	n.d.
Xpot1	108	1*	-	n.d.	n.d.

The increase in solubility of target proteins relative to control cells without co-overexpression of chaperones (set as 1) is given in the column "yield increase". The chaperone combinations for optimal yield of each target protein in the one-step and two-step procedures are indicated. Proteins that exhibited solubility exclusively after chaperone co-overexpression are indicated by "∞". The symbol "*" indicates that no soluble protein was purified from cells with or without chaperone co-overproduction. n.d., not determined. The same protein name can appear more than one time in the list because different constructs (domains or fusions) have been used for its expression.

To further improve the solubility of recombinant proteins we considered that it may be of advantage to allow chaperone-assisted folding in the absence of ongoing protein biosynthesis, thereby preventing the continuous generation of novel aggregation-prone proteins. In one set of experiments we prevented further synthesis of the plasmid-encoded target and chaperone proteins by withdrawal of IPTG. In another set of experiments synthesis of all cellular proteins was blocked by the addition of chloramphenicol (or tetracycline yielding similar results, data not shown) to the culture medium after removal of IPTG.

In 19 out of 34 tested recombinant proteins the two-step procedure resulted in higher solubility yields as compared to the one-step procedure (Table 1). Out of these, two proteins required the two-step procedure for any solubilization, and six proteins showed a chaperone-mediated solubilization only with the two-step procedure. For 13 of the 19 proteins the chaperone combinations 4 or 5 (ELS, KJE, ClpB) resulted in the highest yields of soluble recombinant protein (Table 1). The two-step procedure thus is clearly superior over the one-step procedure in the production of soluble recombinant protein.

**Figure 4:**
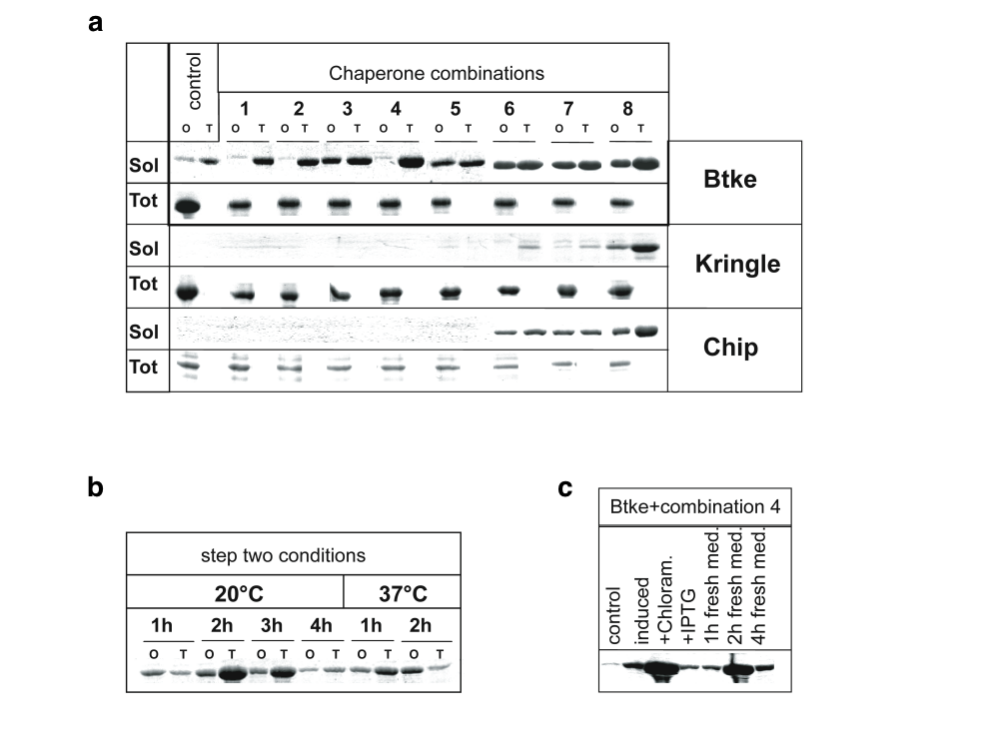
**A two step procedure for successive periods of protein synthesis and folding**. (a) Affinity chromatography purified proteins were separated by SDS-PAGE and stained with SimplyBlue (Invitrogen). Soluble recombinant proteins Btke, Kringle and Chip (Sol) recovered after chaperone-induced folding in BL21(DE3) control cells and cells co-expressing the eight different chaperone combinations described in Fig. 1. Cells were subjected to the one-step (O) or two-step (T) protocols. The total amounts of recombinant protein (Tot) expressed in control and chaperone co-overproducing cells were evaluated after purification of the protein under denaturating conditions. (b, c) Optimization of the (re)folding conditions in step two for Btke using chaperone combination 4. (b) After overnight culturing at 20°C the cells were pelleted, resuspended in fresh medium and cultured for 1 h, 2 h, 3 h, and 4 h at 20°C, or 1 h and 2 h at 37°C in the presence of 200 μg/ml chloramphenicol. For each time point the one-step (O) and two-step (T) procedures were compared. (c) Purified Btke from uninduced cells (control) or cells induced with IPTG according to the one-step procedure (induced) were compared with cells subjected to the two-step procedure with varying conditions: IPTG-induced overnight cultures were pelleted and further grown in fresh media without addition (growth for 1, 2, 4 h) or with addition of chloramphenicol or IPTG (growth for 2 h).

As an example Fig. 4a shows the data obtained for Btke, a domain of the Brutons tyrosine kinase from human, applied to all 5 chaperone combinations (as well as 3 further combinations 6–8 described below). Even in control cells lacking chaperone expression plasmids, the two-step procedure resulted in a slight (3-fold) increase in Btke solubility as compared to the one-step procedure. This is in agreement with the previous observation that in wild type cells, the recovery of soluble recombinant proteins from aggregates can be improved by inhibition of protein biosynthesis [30]. The total amount of Btke produced under chaperone-coexpressing conditions was similar for all 5 strains tested. The one-step procedure resulted in an increased solubility of Btke only for chaperone combinations 3 and 5. In contrast, the two-step procedure, with a two-hour folding period in the presence of chloramphenicol, resulted in increased solubility for all five chaperone combinations, as compared to the control without chaperone co-overproduction. The most striking case was chaperone combination 4 (ELS, KJE, ClpB) in which the solubility of Btke, as compared to the control cells without chaperone co-overproduction, was 42-fold higher for the two-step procedure; in contrast, the one-step procedure (combination 3) yields only a 7-fold increase (Table 1). Interestingly, the optimal chaperone combination differs between the one-step procedure (ELS; combination 3) and the two-step procedure (KJE, ClpB, ELS; combination 4) (Fig. 4a). This switch in chaperone utilization probably reflects the fact that during the (re)folding period in step 2, aggregated Btke is solubilized in a process that requires the concerted action of KJE and ClpB.

Several parameters were found to affect the solubilization efficiency. A two hour incubation of the cells in chloramphenicol containing media at 20°C was optimal for high yields of soluble Btke (Fig. 4b). Longer incubation times or higher temperature lowered the yield, probably because Btke is not entirely stable and is degraded *in vivo *as indicated by the decreased total amounts of Btke found in these cells. Furthermore, significant solubility of Btke (26-fold) could be achieved when transcription of the target gene was decreased by the removal of the inducer IPTG during the folding period, instead of inhibiting protein biosynthesis by chloramphenicol (Fig. 4c).

Although the chaperone combinations described so far showed remarkable effects on the solubility of 34 out of 50 recombinant proteins we aimed at a further optimization of the procedure. Recent findings indicate that the activity of ClpB in disaggregation and refolding of heat denatured proteins is facilitated by the presence of the bacterial sHSPs, IbpA and IbpB (IbpAB) [11]. We tested the effects of co-expression of the *ibpAB *genes (from a high copy number plasmid under pPA1/lacO1 control; pBB572) either alone (combination 6), or with ELS (combination 7) or ELS, KJE, ClpB (combination 8) (Fig. 1).

The co-overproduction of IbpAB (approximately 8–10 fold compared to wild type levels, Fig. 2) resulted in significant further improvement of the solubility of recombinant proteins (Table 2). Remarkably, IbpAB co-overproduction was beneficial even for some of the proteins, which remained completely insoluble with any of the previously tested 5 chaperone combinations. Fig. 4a shows two examples (Chip, Kringle) of such previously insoluble proteins. Out of the 23 proteins tested, the co-overproduction of IbpAB resulted in an increased solubility of 17 proteins in the one-step procedure (7 proteins with combination 6; 3 proteins with combination 7; 7 proteins with combination 8). Using the two-step procedure the yields of 3 additional soluble proteins was increased (Table 2). In sum, 20 out of 23 proteins tested showed increased solubility, and in 12 cases the chaperone co-expression was the only possibility to obtain any soluble protein.

**Table 2:** Effects of IbpAB co-overproduction (combinations 6 to 8) on the yields of purified, soluble target proteins.

Protein	MW (kDa)	Increased solubility by			
		
		one step procedure		two step procedure	
		
		Yield increase	Best chaperone combination	Yield increase	Best chaperone combination
Ag1Ser	30	∞	7	3	7
Chip	87	∞	8	4	8
dLMO	92	∞	6	3	6
Isu2	46	∞	6	3	8
Kringle	27	∞	8	7	8
Luc7	56	∞	8	4	8
Oskar1	16	∞	8	3	8
Ag3Ser	33	∞	6	1	-
Ag4Ser	35	∞	8	1	-
Icy1	45	∞	8	1	-
Oskar4	70	∞	6	1	-
2c18	50	2.5	7	3.5	6
E8R1	85	2.5	6	3	7
Oskar2	115	1*	-	∞	6
Ag2Ser	31	2.5	7	1	-
Synapsin	62	3	6	1	-
1Tep3	47	18	6	1	-
1Tep4	45	6	8	1	-
3x77	44	1	-	3	8
Titin	35	1	-	4	7
Xklp3A3	72	1	-	1	-
Msl1	66	1	-	1	-
NF1	36	1*	-	1*	-

Increases in the amount of purified, soluble protein are given relative to the optimal solubility (set as 1) obtained either by the best of the combinations 1 to 6 tested before (Table 1) or to control cells (combination 4, data not shown). Symbols as in Table 1.

## Conclusion

Taking all results together, the co-overproduction of the entire network of major cytosolic chaperones in *E. coli *cells, combined with a two-step procedure that allows for (re)folding of the recombinant proteins in the absence of ongoing *de novo *synthesis, resulted in an increased solubility of 70% of the 64 recombinant proteins tested. This efficiency is remarkable as most of the constructs used in this study encode proteins that are difficult to be produced in soluble form. The engineered *E. coli *strains and the two-step procedure presented here (patent application No. 10/500,883) should prove particularly useful for biotechnological applications.

## Methods

### Vector description

Plasmids (see Fig. 1a: pBB541, pBB542, pBB550, pBB535, pBB530, pBB540) expressing chaperone genes under the control of the IPTG-regulated promoter PA1/lacO-1 or Plac, and plasmid pBB528 constitutively expressing the LacI^Q ^repressor were constructed as described earlier [28]. Plasmid pBB572 expressing the *ibpA, ibpB *operon under control of the PA1/lacO1 promoter was generated by PCR using primers ON1 (5'-CGGGATCCATATGCGTAACTTTGATTTATCCC-3') and ON4 (5'-GCTCTAGAGCTAGTTAGCTATTTAACGC-3') to amplify the *ibpA, ibpB *operon using chromosomal *E. coli *DNA as template. The PCR product was cut with *BamHI *and *XbaI *and cloned into plasmid pUHE212fdΔ12 [31]. Plasmids encoding various target proteins [see Additional file 1] were delivered from various laboratories to the Protein Expression Unit of the European Molecular Biology Laboratory Heidelberg.

### Transformation procedure

Competent BL21 (DE3) cells (Novagen) were first transformed with a plasmid carrying the lacI^Q ^gene (pBB535, pBB542, pBB550, pBB528) to allow for the controlled expression of chaperones. Subsequently, cells were transformed with appropriate plasmids for selective expression of eight different chaperone combinations and made competent (Fig. 1). Chaperone-overexpressing cells were finally transformed with a plasmid carrying the target gene. Cells were always grown in presence of appropriate antibiotics to ensure the maintenance of all plasmids.

### Protein expression: One-step and two-step procedure

Single colonies from the transformed cells were used to inoculate 3 ml of LB medium supplemented with appropriate antibiotics. Liquid cultures were grown at 37°C until they reached an OD_600 _of 0.4 followed by transfer to 20°C. At an OD_600 _of 0.8 protein expression of plasmid encoded genes was induced by the addition of 0.1 mM IPTG and cells were further incubated overnight at 20°C. For the one-step procedure overnight cultures were pelleted, frozen and stored at -20°C until recombinant proteins were purified. For the two-step procedure overnight cell cultures were pelleted, the medium was removed and the cell pellet was resuspended in the same amount of fresh medium either supplemented with 200 μg/ml chloramphenicol or lacking IPTG. After 2 h incubation of the culture at 20°C the cells were harvested and target proteins were purified.

### Protein purification and evaluation

The frozen bacterial pellet was resuspended in 350 μl of 20 mM Tris HCl buffer (pH 8.0) containing 2 mM PMSF, 0.05% Triton X-100, 1 μg/ml DNase, 5 mM MgCl_2 _and 1 mg/ml lysozyme and incubated on ice for 30 min with periodic stirring. The suspension was sonicated in water for 5 min, an aliquot of 5 μl was withdrawn (homogenate), and the cell debris was pelleted in a minifuge. A 5 μl aliquot of the supernatant was stored and the residual lysate was added to 15 μl of pre-washed magnetic beads (Qiagen) and incubated further for 30 min under agitation before the supernatant was removed. Beads were washed for 30 min with 20 mM K-phosphate buffer (pH 7.8) containing 300 mM NaCl, 20 mM imidazole, 8% glycerol, 0.2%Triton X-100 and subsequently with PBS buffer plus 0.05% Triton X-100. Finally beads were boiled in 12 μl SDS sample buffer and the supernatant loaded on a SDS PAGE. Proteins were detected by Simply Blue Safestain staining (Invitrogen) and the gels were recorded using a Umax Astra 4000U scanner. Protein bands were analyzed using the public NIH Image 1.62f software. Alternatively, the protein was eluted from washed beads using 30 μl PBS buffer plus 0.5 M imidazole and its relative concentration was measured by determination of its absorbance at 280 nm. The folding status was evaluated by circular dichroism using a J-710 spectropolarimeter (Jasco). The proteolytic activity of TEV protease was measured by incubating 500 μg of a fusion protein (GST-Tep1) containing a TEV recognition sequence in its linker in the presence of 5 μg of the TEV protease in Tris-HCl buffer (pH 8), 1 mM EDTA, 1 mM DTT, for 2 hours at 30°C.

## Competing interests

The author(s) declare that they have no competing interests.

## Authors' contributions

A.dM., designed experiments, performed experiments, interpreted data and wrote the manuscript. E.D., A.M., T.T., and B.B., designed experiments, interpreted data and wrote the manuscript.

## Supplementary Material

Additional file 1 **Supplemental tables 1a and 1b**. Click here for file
