# Supplementary material for: Chaperone-based procedure to increase yields of soluble recombinant proteins produced in E. coli
Source: BMC Biotechnol. 2007 Jun 12;7:32. doi: 10.1186/1472-6750-7-32 (PMC1904446; doi:10.1186/1472-6750-7-32)
Supplement: Additional file 1 — Supplemental tables 1a and 1b. [file 1472-6750-7-32-S1.doc]

# SUPPLEMENTARY

**Supplemenatry Table 1a**. Details of recombinantly produced proteins of Table 1A. The noncommercial pETM vectors are described in [1].

| Protein | Entry code | Organism | Cellular  localisation | Vector/ protein construct | Fusion tag  (6xHis + ) |
| --- | --- | --- | --- | --- | --- |
| 1Fringe | NP524126 | D. melanogaster | cytosolic | pETM11/full length | / |
| 2Ap | AAL48762.1 | D. melanogaster | nuclear | pETM11/full length | / |
| 22f21 | AL136604 | H. sapiens | unknown | pETM20/full length | Trx |
| LZip | XP048513 | H. sapiens | cytosolic | pTrcHis/full length | / |
| Oskar3 | A40313 | D. melanogaster | cytosolic | pETM60/full length | NusA |
| 2c18 | P33260 | H. sapiens | unknown | pETM20/full length | Trx |
| 2Tep4 | AgCP8988 | A. gambiae | secreted | pETM20/domain | Trx |
| BtKe | Q06187 | H. sapiens | cytosolic | pETM11/domain | / |
| PhK | Q13393 | H. sapiens | cytosolic | pET3c/full length | / |
| Tep1 | AF291654 | A. gambiae | secreted | pETM11/domain | / |
| Susy | AAL27096 | Z. mays | cytosolic | pETM11/full length | / |
| Xklp3A+Xklp3B | AJ311602 CAA08879 | X. laevis | cytosolic | pST39/domains | / |
| B1R | NP063857 | Vaccinia virus |  | pGEX6P/full length | GST |
| E8R2 | NP063710 | Vaccinia virus |  | pETM60/full length | NusA |
| GTR1 | Q00582 | S. cerevisiae | cytosolic | pTrcHis/full length | / |
| HbpH | NP002473 | H. sapiens | cytosolic | pET9d/domain | / |
| Rolled | NP524763 | D. melanogaster | cytosolic | pETM11/full length | / |
| Xklp3A4 | AJ311602 | X. laevis | cytosolic | pGAT/domain | GST |
| Xklp3B3 | CAA08879 | X. laevis | cytosolic | pETM60/domain | NusA |
| Mash+  Susy | CAA26247 AAL27096 | Z. mays | cytosolic | pST39/full length | / |
| 1Tep3 | AgCP4024 | A. gambiae | secreted | pGEX4T/domain | GST |
| 1Tep4 | AgCP8988 | A. gambiae | secreted | pGEX6P/domain | GST |
| BtKc | Q06187 | H. sapiens | cytosolic | pET3c/full length | / |
| E8R1 | NP063710 | Vaccinia virus |  | pGEX4T/full length | GST |
| MaxF |  | Synthetic |  | pET24d | / |
| Tev prot. | P04517 | Tobacco etch virus |  | pET24d/full length | / |
| Xklp3A1 | AJ311602 | X. laevis | cytosolic | pGAT/domain | GST |
| Xklp3B1 | CAA08879 | X. laevis | cytosolic | pTrcHis/domain | / |
| 2Fringe | NP524126 | D.melanogaster | cytosolic | pGEX6P/domain | GST |
| Xklp3A5 | AJ311602 | X. laevis | cytosolic | pETM20/domain | Trx |
| 2Tep3 | AgCP4024 | A. gambiae | secreted | pETM60/domain | NusA |
| BtKp | Q06187 | H. sapiens | cytosolic | pET3c/domain | / |
| Mash | CAA26247 | Z. mays | cytosolic | pETM11/full length | / |
| PPAT | P23875 | E. coli | cytosolic | pETM11/full length | / |
| Eg5 | XP005889 | H. sapiens | cytosolic | pETM11/domain | / |
| F10L | NP063689 | Vaccinia virus |  | pGEX4T/full length | GST |
| Tep2 | AgCP11437 | A. gambiae | secreted | pETM11/domain | / |
| 1AP | AAL487621 | D. melanogaster | nuclear | pProExHTb/full length | / |
| Chip | NP477082 | D. melanogaster | nuclear | pProExHTb/full length | / |
| dLMO | NP523402 | D. melanogaster | nuclear | pProExHTc/full length | / |
| Pex5P | CAC01120 | H. sapiens | membrane | pETM11/domain | / |
| Endostatin | AAF69009 | M. musculus | secreted | pETM50/full length | DsbA |
| Kringle | NP663779 | H. sapiens | secreted | pETM50/domain | DsbA |
| Lzip2 | AAH25988 | H. sapiens | cytosolic | pET28/full length | / |
| UCP1 | P04633 | R. norvegicus | membrane | pET9d/domain | / |
| Tlc4 | Q9ZD47 | R. prowazekii | membrane | pBAT4/full length |  |
| Xklp3A2 | AJ311602 | X. laevis | cytosolic | pGAT/domain | GST |
| Xklp3A3 | AJ311602 | X. laevis | cytosolic | pGAT/domain | GST |
| Xklp3B1 | CAA08879 | X. laevis | cytosolic | pGAT/domain | GST |
| Xpot1 | AAH20569 | H. sapiens | nuclear | pQE60/full length | NusA |

**Supplementary Table 1b.** Details of recombinantly produced proteins of Table 1b. The noncommercial pETM vectors are described in [1].

| Protein | Entry code | Organism | Cellular  localisation | Vector/ protein construct | Fusion tag  (6xHis + ) |
| --- | --- | --- | --- | --- | --- |
| Ag1Ser | AJ250916 | A. gambiae | secreted | pETM50/domain | DsbA |
| Chip | NP477082 | D. melanogaster | nuclear | pETM50/full length | DsbA |
| dLMO | NP523402 | D. melanogaster | nuclear | pETM60/full length | NusA |
| Isu2 | CAA99445 | S. cerevisiae | unknown | pGEX6P/full length | GST |
| Kringle | NP663779 | H. sapiens | secreted | pETM20/domain | Trx |
| Luc7 | CAA98653 | S. cerevisiae | unknown | pGEX6P/full length | GST |
| Oskar1 | A40313 | D. melanogaster | cytosolic | PETM11/domain | / |
| Ag3Ser | AJ276486 | A. gambiae | secreted | pETM52/domain | DsbA |
| Ag4Ser | AJ276487 | A. gambiae | secreted | pETM52/domain | DsbA |
| Icy1 | NP015073 | S.cerevisiae | unknown | pGEX6P/full length | GST |
| Oskar4 | A40313 | D. melanogaster | cytosolic | pETM60/domain | NusA |
| 2c18 | NP000763 | H. sapiens | unknown | pETM52/domain | DsbA |
| E8R1 | NP063710 | Vaccinia virus |  | pETM60/full length | NusA |
| Oskar2 | A40313 | D. melanogaster | cytosolic | pETM60/domain | NusA |
| Ag2Ser | AJ271117 | A. gambiae | secreted | pETM50/domain | DsbA |
| Synapsin | NP598343 | H. sapiens | cytosolic | pETM20/full length | Trx |
| 1Tep3 | AgCP4024 | A. gambiae | secreted | pGEX6P/domain | GST |
| 1Tep4 | AgCP8988 | A. gambiae | secreted | pGEX6P/domain | GST |
| 3x77 | AL136600 | H. sapiens | unknown | pETM20/domain | Trx |
| Titin | NP653085 | H. sapiens | secreted | pETM20/domain | Trx |
| Xklp3A3 | AJ311602 | X. laevis | cytosolic | pGAT/domain | GST |
| Msl1 | NP476896 | D. melanogaster | nuclear | pETM60/domain | NusA |
| NF1 | BAA02150 | H. sapiens | cytosolic | pETM11/domain | / |

1. A Dummler, AM Lawrence, A de Marco**: Simplified screening for the detection of soluble fusion constructs expressed in E. coli using a modular set of vecto**rs*. Microb Cell Fac*t 2005**,** 4:34.
